# Microglia P2X4 receptors as pharmacological targets for demyelinating diseases

**DOI:** 10.15252/emmm.201809369

**Published:** 2018-07-23

**Authors:** Francesco Di Virgilio, Alba Clara Sarti

**Affiliations:** ^1^ Department of Morphology, Surgery and Experimental Medicine University of Ferrara Ferrara Italy

**Keywords:** Immunology, Neuroscience, Pharmacology & Drug Discovery

## Abstract

Pharmacological activation of the P2X4 receptor expressed by brain microglia may provide a novel avenue to promote remyelination and improve clinical symptoms in experimental autoimmune encephalomyelitis and potentially in multiple sclerosis.

Multiple sclerosis (MS) is a chronic inflammatory disease of the brain and the spinal cord characterized by infiltration of immune cells, gliosis and axonal degeneration (Dendrou *et al*, 2015). Experimental allergic encephalomyelitis (EAE) replicates the typical inflammatory changes in demyelinating lesions of MS and is generally acknowledged as a useful experimental model. The inflammatory infiltrate in AS and EAE is predominantly characterized by mononuclear cells such as microglia, macrophages and lymphocytes. Activation of microglia and blood‐derived macrophages is a main pathogenic mechanism responsible for lymphocyte recruitment and activation, as well as for axonal damage and neurodegeneration. However, a more recent view holds that microglia can also be beneficial for the recovery process in MS by accelerating the removal of myelin debris, by providing neurotrophic factors and by promoting immunosuppression (Miron *et al*, 2013). Beneficial effects of microglia depend on its differentiation towards an anti‐inflammatory phenotype.

Microglia, like tissue macrophages, is thought to be present in two alternative activation states, pro‐inflammatory type I or classically activated (M1), and anti‐inflammatory type II, or alternatively activated (M2). Nowadays, this distinction in microglia is believed to be not as clear‐cut as in macrophages, since microglia activation is a highly dynamic and plastic process where the transition from one activation state to the other occurs along a continuum of functional changes (Wolf *et al*, 2017). Nevertheless, the identification of a pro‐inflammatory or an anti‐inflammatory microglia phenotype offers a useful operational framework for decoding microglia responses and understanding its participation in disease processes.

Microglia activation and graded differentiation towards a pro‐inflammatory or anti‐inflammatory phenotype are driven by factors released from injured nerve cells (damage‐associated molecular patterns, DAMPs) or pathogen‐derived factors (pathogen‐associated molecular patterns, PAMPs), but receptors and intracellular pathways involved are poorly understood. It would be useful to be able to direct microglia differentiation towards one or the other differentiation pathway, but how this might be achieved is as yet unknown. This is an important issue because while pro‐inflammatory microglia is thought to be a prime culprit of immune cell recruitment and brain damage, anti‐inflammatory microglia releases neuroprotective factors, dampens immunity and supports nerve cell recovery.

Several DAMPs are released in the CNS following stress, trauma and infection: aggregated peptides, misfolded proteins, nucleic acids, nucleotides. In particular, it is now clear that intracellular nucleotides, mainly ATP, are released early following CNS stress or injury (Di Virgilio *et al*, 2009). The sudden increase in the local extracellular ATP concentration is perceived as an unequivocal signal of cell injury and as a pro‐inflammatory stimulus. Microglia express a panoply of receptors for extracellular nucleotides named P2 receptors (P2Rs) and further classified into metabotropic P2YRs and ionotropic P2XRs. P2Rs have been variably associated with neuroinflammation, with most attention being focused on the P2X7R subtype, a potent stimulator of the NLRP3 inflammasome and of the IL‐1β processing and release (Kettenmann *et al*, 2011; Di Virgilio *et al*, 2017). In the P2XR subfamily, the P2X4R has an established role in the pathogenesis of neuropathic pain (Inoue & Tsuda, 2018), but in other respects, its involvement in neuroinflammation has been mainly anecdotal. In neuropathic pain, the P2X4R is upregulated and, upon stimulation with ATP, drives the release of the trophic factor, brain‐derived neurotrophic factor (BDNF). In macrophages, but not in microglia, the P2X4R has been associated with the prostaglandin E2 (PGE2) release and with the initiation of inflammatory pain (Ulmann *et al*, 2010). The P2X4R has an unusual cellular distribution as it is predominantly associated with the lysosomal compartment, where it may control lysosome–phagosome fusion and more generally lysosome trafficking (Huang *et al*, 2014). In this function, the P2X4R has a key role in the release of surfactant from lung alveolar type II epithelial cells (Fois *et al*, 2018). Lysosomes contain high Ca^2+^ concentrations that may be released into the cytoplasm via P2X4R, and therefore activate multiple intracellular signal transduction pathways (Murrell‐Lagnado, 2018). Thus, both plasma membrane‐expressed and lysosome‐localized P2X4Rs might be responsible for the modulation of microglia responses elicited by DAMP or PAMP stimuli.

In their study, Zabala *et al* (2018) highlight a novel and essentially counterintuitive function of microglia P2X4R as an anti‐inflammatory and neuroprotective receptor. In a model of acute EAE triggered by immunization with myelin oligodendrocyte glycoprotein (MOG), these authors found an increased expression of *P2x4r* at the disease peak and throughout the recovery phase. Overexpression of *P2x4r* was paralleled by upregulation of interferon regulatory factors 8 (IRF8) and 5 (IRF5), two transcription factors previously shown to induce P2X4R expression in microglia. Since microglia is activated and injured during the early stages of EAE, thus releasing factors that drive the sustained recruitment of blood‐derived inflammatory cells, and microglia damage and demise can be prevented by P2X4R blockade, it is anticipated that P2X4R blockade would prevent amplification of inflammation and ameliorate EAE. On the contrary, and quite surprisingly, Zabala *et al* ((2018) found that P2X4R blockade with the semi‐selective antagonist TNP‐ATP exacerbated EAE. Accordingly, EAE had a worse course in P2X4^−/−^ versus P2X4 WT mice, and TNP‐ATP treatment had no effect in the absence of P2X4R. In addition, P2X4R blockade did not affect the efficacy of immunization by MOG, T‐cell proliferation or immune cell infiltration in brain and spinal cord, suggesting that lack of P2X4R activity did not affect the overall process of immunization. Gene expression profiling showed that P2X4R blockade significantly increased pro‐inflammatory gene expression during the recovery phase of EAE, while anti‐inflammatory genes were unaffected. By using as markers of microglia polarization the mannose receptor (anti‐inflammatory) and the inducible nitric oxide synthase (iNOS, pro‐inflammatory), the authors further showed that P2X4R‐inhibition promoted a microglia polarization towards a pro‐inflammatory phenotype. Since the mechanism by which microglia switch from a pro‐inflammatory to an anti‐inflammatory phenotype is crucial during the recovery phase of MS, the role of P2X4R in oligodendrocyte differentiation and remyelination was investigated. Oligodendrocytes lack the P2X4R therefore they are intrinsically insensitive to manipulations of P2X4R expression and function. However, the culture of oligodendrocyte precursors in the presence of microglia‐conditioned media showed that oligodendrocyte differentiation was more efficiently promoted by the medium conditioned by anti‐inflammatory versus pro‐inflammatory microglia, and this effect could be blocked by microglia treatment with TNP‐ATP. The key role of microglia P2X4R in supporting oligodendrocyte differentiation and production of myelin basic protein depended on the release of BDNF, an agent known to support oligodendrocyte differentiation and myelination. The P2X4R blockade also severely affected the removal and degradation of myelin debris by microglia, a process extremely important in the regenerative response (Fig 1).

**Figure 1:**
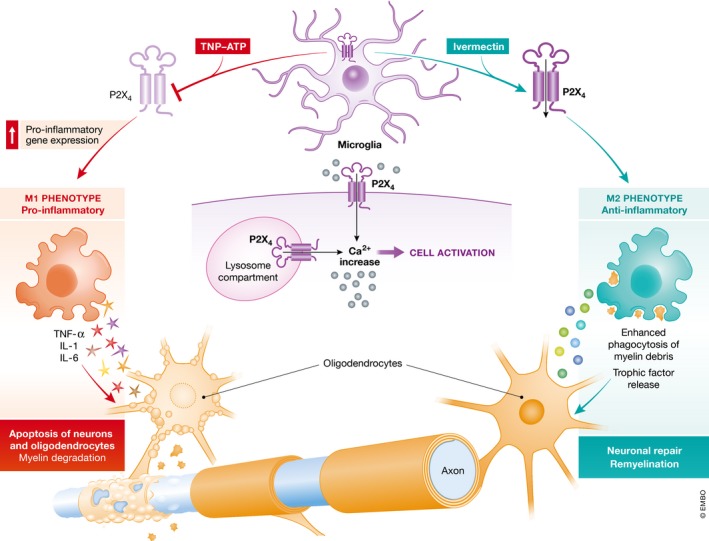
Modulation of the P2X4 receptor promotes pro‐ or anti‐inflammatory microglia differentiation Inhibition of the P2X4 receptor (P2X4R) by the semi‐selective blocker TNP‐ATP (red arrow) drives microglia differentiation towards a pro‐inflammatory (M1) phenotype characterized by the enhanced release of pro‐inflammatory cytokines. On the contrary, P2X4R activation by ivermectin (IVM) (green arrow) drives differentiation towards an anti‐inflammatory phenotype characterized by trophic factor release and enhanced phagocytic activity. M1 microglia exacerbates axonal degeneration, while M2 microglia accelerates neuronal repair. The P2X4R is localized both on the plasma membrane and on the lysosomal membrane; therefore, the diverse responses coupled to its inhibition or activation, respectively, may depend on the differential stimulation of the P2X4R on these cellular compartments.

Novel and more effective therapies for MS are urgently needed. To investigate the potential of P2X4R as a drug target in MS, Zabala *et al* (2018) investigated the effect of ivermectin (IVM), a positive allosteric modulator of the P2X4R (Khakh *et al*, 1999) approved by FDA for the treatment of parasitic diseases. IVM administration to EAE mice induced a significant amelioration of motor deficits and corticospinal functions and promoted polarization of microglia towards an anti‐inflammatory phenotype, enhanced endosome–lysosome fusion and accelerated remyelination. IVM did not potentiate these functions in P2X4^−/−^ mice.

This study highlights the complex role of the P2X4R in multiple microglia responses: pro‐inflammatory versus anti‐inflammatory polarization, the release of neurotrophic factors, phagocytosis of myelin debris. It is likely that such diverse functions depend on the localization in different cellular compartments, the plasma membrane and the lysosomal compartment, a feature that likely endows P2X4R with the ability to activate multiple intracellular pathways. Given that ATP is an ubiquitous extracellular messenger and P2 receptors are expressed by virtually all inflammatory cells, findings by Zabala *et al* (2018) raise the intriguing question as to whether pathophysiological responses associated with the stimulation of other members of the P2R family may also depend on the activation of intracellularly located versus plasma membrane‐expressed receptors, or on a cross‐talk between receptors located in different cellular compartments. Finally, and more importantly, this study may open the pathways to the development of novel, P2X4R‐targeted, drugs (IVM) for the treatment of MS.

## Conflict of interest

FDV is a member of the Scientific Advisory Board of Biosceptre Ltd, a UK‐based biotech company involved in the development of P2X7R‐targeted therapeutics. ACS declares no conflict of interest.
